# Predictor role of marital conflict on maternal competency with mediating role of perceived stress and concerns during pregnancy: A structural equation model

**DOI:** 10.1002/npr2.12309

**Published:** 2023-01-24

**Authors:** Sepideh Mohammad Alipour, Mitra Rahimzadeh, Zahra Mehdizadeh Tourzani, Zohreh Mahmoodi, Sara Esmaelzadeh Saeieh

**Affiliations:** ^1^ Alborz University of Medical Sciences Karaj Iran; ^2^ Social Determinants of Health Research Center, School of public health Alborz University of Medical Sciences Karaj Iran; ^3^ Instructor of Midwifery Alborz University of medical science Karaj Iran; ^4^ Social Determinants of Health Research Center Alborz University of Medical Sciences Karaj Iran

**Keywords:** concern, life stress, marital conflict, maternal role, pregnancy

## Abstract

**Aim:**

Pregnancy prepares the mother for the transition to motherhood. Maternal concerns during pregnancy cause reduced time spent with the spouse and lack of social support; additional stresses such as marital conflicts could impact maternal competency. This study aimed to assess the predictive effect of marital conflicts with the mediating role of perceived stress and pregnancy concern on maternal competency.

**Methods:**

This was a longitudinal study. It was done on 250 mothers referred to selected health centers in Alborz province. The sampling method was convenient. Marital conflicts, perceived stress, and pregnancy concerns questionnaires were completed in the third trimester of pregnancy, and the maternal competency questionnaire was completed 6 weeks after delivery. Data were analyzed by SPSS software and smart partial least squares.

**Results:**

The results of structural equations showed that marital conflicts have a negative and significant effect on maternal competency (*β* = −0.14), marital conflicts have a positive and significant effect on perceived stress (*β* = 0.42), and marital conflicts have a positive and significant effect on pregnancy concern (*β* = 0.31). Also, perceived stress negatively and significantly affected maternal competency (*β* = −0.36).

**Discussion:**

Results of the study showed the necessities for screening and identifying mothers with conflicts and assessing the perceived stress of mothers will improve the mental health of pregnant mothers and consequently increase maternal competency.

## Abstract 2

We prospectively described relationship between marital conflict, stress and concern during pregnancy and maternal competency. Results showed that three variables predicted 23% of maternal competency variance.

## Graphical abstract

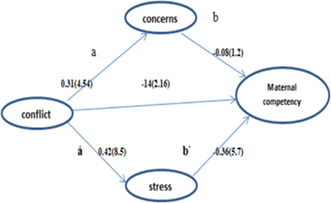

## 1 INTRODUCTION

Pregnancy is a series of transient events that involve physiological, physical, and psychological changes in a woman's life. The perinatal period is often very challenging and requires constant psychosocial adjustment of parents.^1^ New responsibilities and maternal transitions have high‐stress levels and psychological problems in mothers. Results of a study have shown the impact of pregnancy stress on maternal competency, child growth, and development.^2^

### 1.1 Maternal competency

Maternal competency is a process in which the mother achieves competence in the role and integrates the mothering behaviors into her established role so that she feels comfortable with her identity as a mother.^3^

Transition to motherhood is an important stage in women's lives and adds many responsibilities and roles to their lives.^4^ Developing a way to be good and achieve maternal competency is essential in adapting to the maternal role.^5^

The formation of maternal competency is a progressive process that begins at the beginning of pregnancy and continues until the postpartum period.^6^

Competency in the maternal role should increase with time and as the woman's self‐concept develops. Skill and sensitive care will be provided with maternal competence that responds to infants' needs and fosters infant development.^7^ The effective factors for maternal competency were intimate partner support, social support, maternal perception of infant behavior, and mental health.^8^

The transition to motherhood is not only associated with positive experiences but also with conflict and despair.^9^ Furthermore, the dissatisfaction with the maternal experience of early motherhood, if not appropriately taken, preserves and negatively impact postnatal mood and caregiving behavior.^10^ Identifying indicators that best predict maternal competency can be necessary for the support of mothers in the prenatal period to avoid adverse outcomes for mothers and infants.^11^

### 1.2 Marital conflicts and maternal competency

Marital adjustment is one of the factors that cause mothers to adapt to the complex maternal process after childbirth and help increase maternal competency.^12^ However, some women may not be able to cope with the physiological changes of pregnancy for reasons such as not having adequate support, which can lead to disputes over child care and marital conflicts. Marital conflicts can be an essential factor affecting the degree of competency of primiparous mothers to the fetus. Marital relationship improves motherhood transition directly and recovers relationships with relatives, so organizing marital relationships during pregnancy and investigating the quality of the relationship of which are essential.^13^

### 1.3 Marital conflicts and perceived stress

Couples' relationships and emotional support are related to lower levels of Perceived stress. Inversely, marital conflict or domestic violence is predictive of high levels of perceived stress. Parents with marital conflicts experienced fewer partners' support, more significant perceived stress, poor health, lower comfort, and more somatic and psychological symptoms.^14^ In addition, results of a study showed that marital conflicts are among the factors that cause stress experiences and, consequently, the Immunodeficiency system during pregnancy.^15^

### 1.4 Marital conflicts, pregnancy concerns, and maternal competency

Pregnancy‐specific concerns cause pregnancy complications. During pregnancy, the severity of concern and anxiety appears to be U‐shaped, so in the first and third trimesters, the level of concern and anxiety is higher than in the second trimester.^16^ Results of studies showed that the most worries about pregnancy are related to childbirth, child health, and maternal competency.^17^, ^18^ Pregnancy could be a burden due to a lack of social support and additional stresses, such as family conflicts.^19^ A study showed that nullipara mothers reported higher levels of conflict with spouses compared with their childless life. Concerns about their family life, relationship with their partner, and fears about future conflicts could impact maternal competency.^20^ And also, the results of the study showed that mothers' adaptation was lowest in martially with conflicts.^14^ Worries during pregnancy about socio‐medical, relationship, reproductive loss, health, and socio‐economic problems also influenced maternal emotional functioning, contributing to higher distress levels.^21^

### 1.5 Perceived stress during pregnancy and maternal competency

Perceived stress arise based on the dissatisfied relationship between parents.^5^ A study's results have shown a significant relationship between perceived maternal stress and marital conflicts. The results of a study have shown that perceived stress during pregnancy affects the quality of marital relationships.^22^ Also, a longitudinal study on the first parents showed that men and women reported a low adjustment rate during pregnancy.^23^ A study showed a positive relationship between the psychological state of the couple (in terms of perceived stress, depression, and anxiety) and the rate of marital conflict during pregnancy.^22^

Parents in the postpartum period are required to learn new roles, have a sense of responsibility in the family, create a safe family environment for the infant, and take care of him/her. Mothers adapt to their new role by establishing a proper bond with their baby, breastfeeding, and learning coping strategies.^24^

**FIGURE 1:**
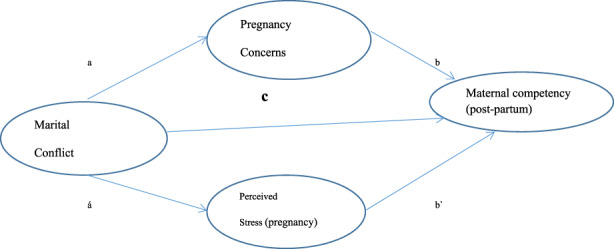
Conceptual model

The prenatal period is critical due to its significant implications for maternal competency. Maternal concerns during pregnancy cause reduced time spent with the spouse and lack of social support; the presence of additional stresses such as marital conflicts could impact maternal competency. There is a need to study marital conflict in the transition to motherhood. The present longitudinal study aimed to Predict role of marital conflict on maternal competency with mediating role of perceived stress and concerns during pregnancy (Figure 1) and following conceptual model, our hypothesis of study were: (a) “Marital conflicts during pregnancy affect directly on the perceived stress during pregnancy.” (b) “Perceived stress during pregnancy would mediate the direct associations between marital conflict during pregnancy and maternal competency after childbirth”. (c)“ Marital conflicts during pregnancy affect directly on the maternal competency after childbirth.” (á) Marital conflicts during pregnancy affect directly on the Pregnancy concern during pregnancy.” (b) “Pregnancy concern would mediate the direct associations between marital conflict during pregnancy and maternal competency after childbirth”.

## 2 METHOD

This study was a prospective longitudinal study approved by the Ethics Committee of Alborz University of Medical Sciences with code (IR.ABZUMS.REC.1398.249). Participants were 250 pregnant mothers that referred to selected health centers in Alborz province, which had the most patients. Participants received information about the study, and the consent form was provided via e‐mail or social media. They were free to decline participation or withdraw at any study time. The Helsinki guideline performed all ethical considerations. Firstly, the list of pregnant mothers was prepared, and then, based on their eligibility, the mothers were selected.

Participant's inclusion criteria were: being in the third trimester of pregnancy (gestational age more than 28 weeks), had age between (20–40) years, being married, singleton pregnancy, Nulliparity, exclusion criteria include: Having neurological and mental illnesses, Having pregnancy complications, addiction, Occurrence of stressful events (such as the death of relatives, etc. in the last 6 months), having a baby with a diagnosed anomaly. The questionnaire was prepared in Google form format and distributed using social networks. Also, if the mothers did not have access to social networks, it would be completed with prior coordination in receiving postpartum care.

The sampling method was convenient. The total number of questions in the study was 76 items; Munro suggested 3–5 subjects for each question^25^; in this study, three subjects were selected on average for each question, and the total sample size in this study was 228 people, with a 20% dropped out of sampling 272 people was estimated. However, 22 participants could not be accessed after delivery due to the longitudinal nature of the study. Moreover, a sample size of over 200 is satisfactory for the conduction of Structural Equation Modeling (SEM).^26^

## 3 QUESTIONNAIRES

### 3.1 Marital conflict questionnaire (MCQ)

This questionnaire is a 42‐item tool designed to measure marital conflict by Sanaei Zaker. This instrument measures seven dimensions of marital conflict and answers in the form of a 5‐point Likert scale ranging from (never = 1 to always = 5). In this tool, a higher score means more conflict. A reliability coefficient for the whole questionnaire was obtained as 0.80 using alfa‐Cronbach's, and consistency reliability was 0.92 through test retest, which suggests its appropriate reliability in the Iranian community.^27^ Isanejad et al. reported the questionnaire's reliability (Cronbach's coefficient = 0.93).^28^ Since the present study was performed on nulliparous women, the dimensions of child support (7 questions) were removed. The validity of the questionnaire was determined through construct validity with partial lease squares confirmatory factor analysis, and nine items with factor loading less than 0.7 were removed from the questionnaire; the reliability coefficient was determined again through Cronbach's alpha test. It was above 0.9 for each dimension and above 0.92 for the total scale.

### 3.2 Prenatal distress questionnaire (PDQ)

This questionnaire was developed by Aldercine and Lynn.^29^ This questionnaire has three subscales of concern about birth and baby, concern about weight and body image, and concern about emotions and relationships that have been used to measure pregnancy‐specific concerns. The psychometric of the questionnaire was assessed by Yousefi et al.^30^ In Iran, and the internal consistency for the total questionnaire was calculated to be 0.74, suggesting the appropriate reliability of the questionnaire and consistency reliability was 0.85 through a test retest.

### 3.3 Perceived stress scale (PSS)

Cohen et al.^31^ designed this questionnaire. It has 14 questions on the five‐point Likert scale. The overall score indicates perceived stress. Safaei and Shokri^32^ have reported Internal consistency (Cronbach's coefficient = 0.76) and test–retest reliability 0.81 for total perceived stress.

### 3.4 Parenting sense of competence scale (PSOC)

Gibaud‐Wallston^33^ designed PSOC, which contains 17 items with a five‐point Likert scale (1 – completely disagree, 5 – completely agree), in which the higher scores represent higher parental competency. Esmaelzadeh has reported Cronbach's alpha of (0.78) and test–retest reliability 0.78 for a total scale.^34^

### 3.5 Data analysis

In the present study, marital conflict was the independent variable, maternal competency was the dependent variable, and pregnancy concern and perceived stress were mediating variables (Table S1). Data were entered into SPSS‐16 software (Table S2), and then the normality of the data was determined by skewness and kurtosis. Missing data was determined and replaced with the median. Finally, Pearson correlation tests were used to analyze the data. Also, Smart PLS3 software was used to determine the validity and reliability of the model as well as the relationship between variables in the model. Smart PLS empowers the researcher to execute complex models and is implemented in the measurement model (external model) and structural model (internal model). First, confirmatory factor analysis was performed, and the model validity and reliability, model fit, and quality of the model were determined. Finally, the relationships between variables were determined by structural equations.

## 4 RESULTS

The results showed that 96 (38.4%) pregnant mothers were between 20 and 30 years old. The study showed that 148 (58%) mothers were housewives. 90 (66%) had postgraduate and bachelor's degrees. 152 (60.8%) pregnant women had a vaginal delivery. 132 (56.5%) of the neonates were male. 238 (95.2%) infants were breastfed. Fourteen (5.6%) infants were admitted to the intensive care Unit. The demographic characteristics of participants are mentioned in Table 1.

**TABLE 1:** Demographic characteristic of participants

Age		*F* (%)	Marital duration (year)		*F* (%)
	<20	45 (18)		<1	73 (29.2)
	20–30	96 (38.4)		1–3	84 (33.6)
	30–40	76 (30.4)		3–5	62 (24.8)
	<40	33 (13.2)		>5	31 (12.4)
Occupation			Type of delivery		
	Housewife	148 (58)		NVD	152 (60.8)
	Employee	102 (42)		CS	98 (39.2)
Education level			Breast feeding	Yes	238 (95.2)
	High school	23 (9)		No	12 (4.8)
	Diploma	61 (25)			
	University	166 (66)	Baby gender		
				Boy	143 (56.5)
Neonatal hospitalized				Girl	110 (43.5)
	Yes	14 (5.6)			
	No	236 (94.4)	Weigh of neonate	Mean ± SD	3.20 ± 413

The correlation matrix results showed a significant relationship between marital conflict and perceived stress, pregnancy concern, and maternal competency (Table 2).

**TABLE 2:** Correlation matrix of marital conflict, perceived stress, pregnancy concern, and maternal competency of pregnant mothers

	Mean ± SD	1	2	3	4
1. Marital conflicts	149.5 ± 20.4	1			
2. Concerns during pregnancy	13.3 ± 7.4	0.31*	1		
3. Perceived stress during pregnancy	32.14 ± 8.17	0.39*	0.4*	1	
4. Maternal competency	61.13 ± 11.14	0.35*	0.26	0.47*	1

*Note*: *p < 0.01. *Pearson's test.

### 4.1 Measurement model

The measurement model in PLS confirmatory factor analysis examined internal consistency reliability, convergent validity, and discriminant validity. The first test is the homogeneity test, which is the confirmatory factor analysis, and questions with a factor load of less than 0.7 were eliminated from the model.^35^

Cronbach's alpha, Composite reliability, and Spearman RHO‐A correlation tests were used for model reliability. In all cases, values greater than 0.7 were acceptable^36^ (Table 3).

**TABLE 3:** Criterion of reliability of the model

Variables	Cronbach's alpha	Composite reliability	*RHO_A*
Marital conflicts	0.93	0.94	0.93
Pregnancy concern	0.83	0.87	0.84
Perceived stress	0.84	0.87	0.84
Parental competence	0.70	0.79	0.7

Results confirmed the model's reliability based on Cronbach's alpha, Composite reliability (CR), and Spearman RHO‐A correlation tests.

The average variance extracted (AVE) was used to determine the convergent validity of the model. In this test, the AVE of each variable was higher than 0.5, and in the second convergent validity test, CR for each variable was higher than the AVE of the same variable.^37^ The results showed that the model had good convergent validity. The results showed that the model had good convergent validity.

The Cross‐loading test, Fornell‐Larcker, and Hetro trait mono trait (*HTMT*) criterion were used to determine discriminant validity.^38^ In the table of cross‐loading, each question had the highest factor load for the determining variable, and the results showed that the questions of each variable are divergent or different from the items of the other variables.

The Fornell‐Larcker test results showed that the square root AVE of all variables is greater than the correlation of that variable with other variables (Table 4). And also HTMT method showed the correlation between the two constructs to be less than 0.9. Therefore, the discriminant validity of the variables is also confirmed.

**TABLE 4:** *Fornell‐Larcker* criterion, (*CV COM*) index and *SRMR* index of model

	Marital conflicts	Concern	Perceived stress	Parental competence	*CV COM*	*SRMR*
Marital conflicts	0.61				0.31	0.079
Concern	−0.31	0.70			0.32	
Perceived stress	−0.42	0.43	0.64		0.28	
Parental competence	0.59	0.45	−0.28	0.59	0.15	

To determine the quality of the external measurement model in terms of predictive power was used crossed validated communality (CV COM) index, Henseler^39^ compared this index with three numbers, 0.02, 0.15, and 0.35, and represents mild, moderate, and good quality, respectively. In this study, CV COM values for all variables were more than 0.15 (Table 4). The measurement model has a moderate predictive rate.

### 4.2 Internal structural model

**FIGURE 2:**
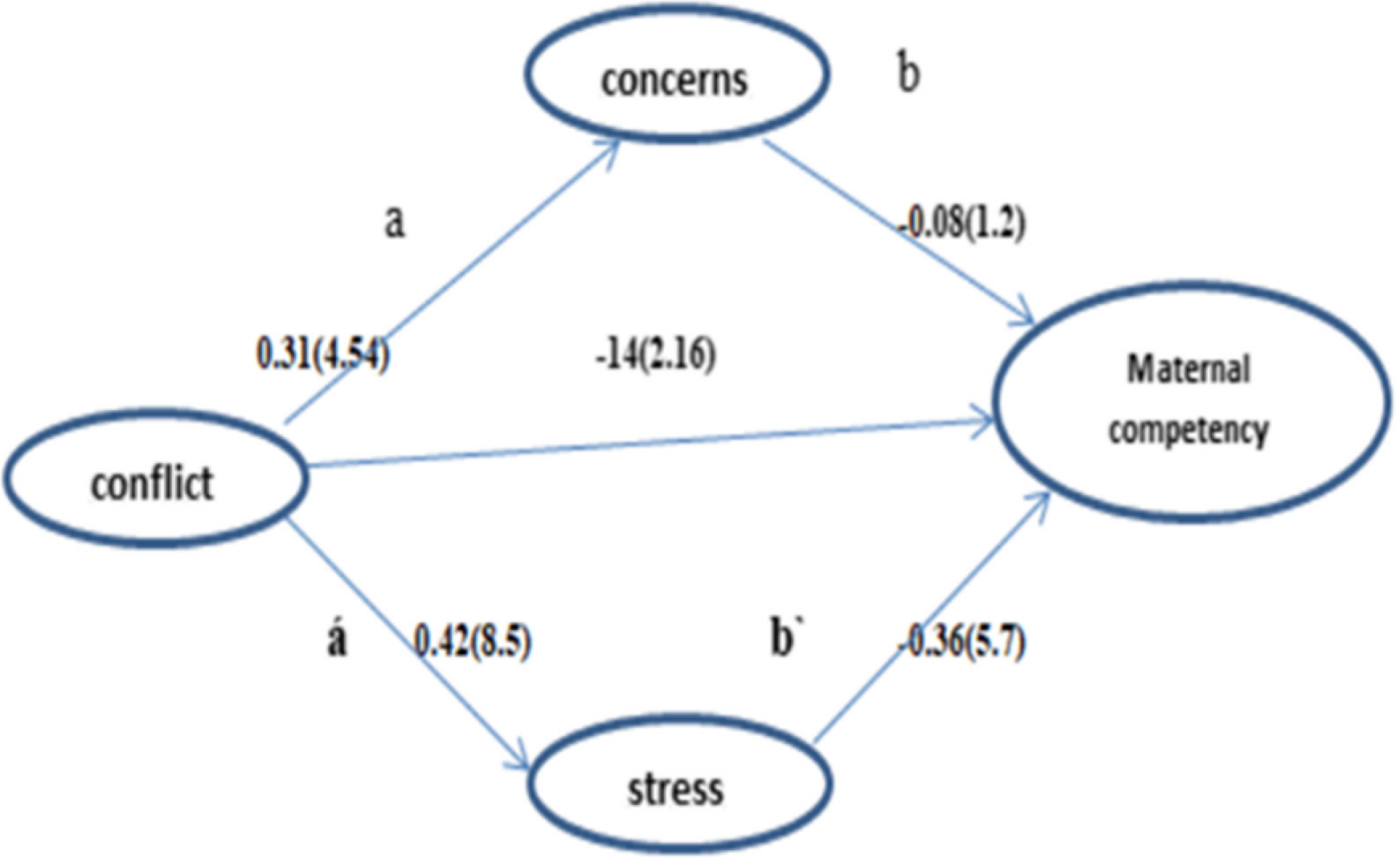
Partial least squares‐path coefficient

Structural model evaluation was used to determine the relationship between the variables of marital conflict, perceived stress, pregnancy concern, and maternal competency. Figure 2 and Table 5 show the significant coefficients and path coefficients of the research variables (Table 5).

**TABLE 5:** Path coefficient and determined variance of structural equation model

Hypothesis	β	*T* value	*p* value	Result
Conflict→maternal competency	−0.14	2.16	0.03	Accepted
Conflict→stress	0.42	8.5	0.001	Accepted
Conflict→concern	0.31	4.54	0.001	Accepted
Stress→maternal competency	−0.36	5.7	0.001	Accepted
Concern→maternal competency	−0.08	1.2	0.2	Reject
Maternal competency	*R* ^2^		*R* adjusted	
	0.23		0.22	

Results of the study showed that marital conflicts have a negative and significant effect on maternal competency (β = −0.14), marital conflicts have a positive and significant effect on perceived stress (β = 0.42), marital conflicts have a positive and significant effect on pregnancy concern (β = 0.31). Also, perceived stress negatively and significantly affects maternal competency (β = −0.36).

According to the value of R square, the three variables of marital conflict perceived stress, and pregnancy concern together predict 23% of maternal competency. This value is a moderate value based on the three values of Chin.^40^ 0.19, 0.33, and 0.67 Also, to determine the fit of the model, the Standardized Root Mean Square Residual (*SRMR*) index was used, which according to Hensler, it should be less than 0.08,^41^ and in this study, it was 0.079, which showed that the model has a good fit.

For mediator analysis, the Sobel criterion was used.^42^ The model was implemented once without perceived stress and concerns variables (zero model). The direct path(C) marital conflicts on maternal competency were significant (Figure 1). To enter the mediator of pregnancy concern, the second indirect path (b) (the effect of pregnancy concern on maternal competence) was insignificant; therefore, pregnancy concern wasn't a mediator. However, with the entry of perceived stress into the model, the first indirect path of marital conflict on perceived stress was significant (á), and also the second indirect path of perceived stress on maternal competence was significant (b^/^) (Figure 1). The variance accounted for was calculated (*VAF =* 0.59), and based on the Sobel criterion, *VAF* between 0.2–0.8 is considered a partial mediator.

## 5 DISCUSSION

The results of the study showed that marital conflicts negatively affect maternal competency. If the rate of marital conflicts changes by one unit, it has an inverse effect on maternal competency by 0.14. Altiparmak et al. showed that adjustment in marital relationships positively and significantly impacts maternal and fetal attachment behavior and breastfeeding self‐efficacy.^24^ In a study, Mutla et al. indicated that marital adjustment increases maternal attachment to the fetus.^43^ Studies have also shown that the relationship between mother and husband affects the attachment behavior of mother and fetus. Mothers who have problems in their marital relationship also have difficulties in behavior with their children; a healthy marital relationship is essential to have competency for the mother.^44^ The study has shown that women are more affected by marital conflicts than men, which is more effective in accepting the maternal role because the mothers feel pregnancy and its challenges more..^45^ the results of studies have shown that social support is an essential factor in improving maternal competency.^46^, ^47^ In addition, the spouse has the most supportive role for mothers in accepting the maternal role, so improving marital relationships increases the acceptance of maternal competency.^48^

The study's results showed that perceived stress affects maternal competency and if the perceived stress variable changes by one unit, maternal competency changes in the opposite direction by 0.36. Therefore, perceived stress has a negative and significant effect on maternal competency. In a study, Chang et al. found that mental health during pregnancy (depression and anxiety) was inversely related to child development in the first 6 months after birth.^49^ Other studies have indicated that women with postpartum depression are less self‐efficacy in parenting activities and perform fewer childcare activities, which causes delays in the child's development. Maternal stress and mental health play an important role in maternal competency and child development.^50^

The results of the present study showed that marital conflicts have a positive and significant effect on pregnancy concerns. If marital conflicts change one unit, it changes 0.31 in the same direction. Pregnancy prepares the mother for the transition to motherhood. Maternal concerns during pregnancy cause reduced time spent with the spouse, making it difficult to do housework and restricting leisure time, which is associated with more conflicts.^51^ On the other hand, exposure to screening tests, sonography, and glucose tolerance test during pregnancy will further increase the mother's concern.^52^ As the initial transition to motherhood begins with pregnancy, there are concerns such as accepting new social roles, restrictions on freedom, and spending more time, energy, and budget, the quality of marital relationships affect these concerns during pregnancy. As a result, maternal concerns further increase marital conflicts.^53^

The study showed that pregnancy concern does not affect maternal competency. However, since the present study measured maternal competence prospectively 6 weeks after delivery, and in this study, pregnancy‐specific concerns were examined, so the trend of time and initiation of the maternal transition process may reduce the impact of pregnancy‐related problems and concerns on maternal competency.

Results of the study showed that perceived stress partially mediated the correlation between marital conflict and maternal competency. In addition, a study showed a significant positive correlation between parental attachment, parenting competency, parenting stress, and prenatal mood mediated the association between competency and parenting stress regarding the marital relationship.^5^

According to the results, focusing on mental health, concerns during pregnancy, and improving communication skills that increase the level of intimacy within couples and the psychological wellbeing of pregnant women could impact maternal competency.

This study suggested that designed interventions for mothers to maintain a more mental stance by increasing the relationship between them and their husbands, decreasing perceived stress and concerns for improving maternal competency.

One of the study's limitations was the self‐reporting of individuals, which may lead to a bias in the provision of information. On the other hand, the value of the variance of maternal competency described by the three variables of pregnancy concern perceived stress and marital conflict was 23%, which necessitates further examining other variables in predicting maternal competency.

## 6 CONCLUSION

This study showed the relationship between marital conflict, perceived stress, and pregnancy concern with maternal competency. Also, it explored mediated role of perceived stress in the relationship between marital conflict and maternal competency. Therefore, planning is needed to provide interventions for improving the quality of marital relationships during pregnancy. Besides, screening and identifying mothers with conflicts and assessing the perceived stress of mothers will improve the mental health of pregnant mothers and consequently increase maternal competency.

## AUTHOR CONTRIBUTIONS

SES and SMA conceived and designed the study. SMA performed the study. MR analyzed data. SES, SMA, ZM, and ZMT wrote the primary draft of the paper. All authors read and approved the final version of the manuscript.

## FUNDING INFORMATION

The study is extracted from the first author's master's thesis in midwifery, which was financially supported by Alborz University of Medical Sciences in Iran.

## CONFLICT OF INTEREST

The authors declare that they have no conflict of interest.

## ETHICAL APPROVAL

The study was approved by the Ethics Committee of Alborz University of Medical Sciences with code (IR.ABZUMS.REC.1398.249). The study was conducted following the Helsinki declaration. The participants received written and oral information about the study and written informed consent was obtained from them. They were free to decline participation or withdraw at any time.

## Supporting information


**Table S1.** Variable information

**Table S2.** Data information

## Data Availability

The data that support the findings of this study are available in Supporting information.
