# Supplementary material for: Predictor role of marital conflict on maternal competency with mediating role of perceived stress and concerns during pregnancy: A structural equation model
Source: Neuropsychopharmacol Rep. 2023 Jan 24;43(1):95–102. doi: 10.1002/npr2.12309 (PMC10009430; doi:10.1002/npr2.12309)
Supplement: Supplementary file 1 — Table S1. Variable information [file NPR2-43-95-s001.pdf]

Variable Information

Variable	Position	Label	Measurement Level	Role	Column Width	Alignment	Print Format	Write Format
id	1	<none>	Scale	Input	11	Right	F40	F40
age	2	<none>	Nominal	Input	9	Right	F25	F25
job	3	<none>	Nominal	Input	5	Right	F15	F15
education	4	<none>	Nominal	Input	7	Right	F35	F35
husband.age	5	husband age	Nominal	Input	5	Right	F27	F27
husband.education	6	husband education	Nominal	Input	5	Right	F35	F35
husband.job	7	husband job	Nominal	Input	12	Right	F12	F12
income	8	income	Nominal	Input	10	Right	F32	F32
marraige.lenght	9	marriage duration	Nominal	Input	14	Right	F24	F24
N.abortion	10	abortion	Nominal	Input	6	Right	F6	F6
GA.w	11	gestational age(week)	Nominal	Input	6	Right	F40	F40
infertility	12	infertility history	Nominal	Input	11	Right	F6	F6
desease.history	13	history of disease	Nominal	Input	19	Left	A79	A79
m1	14	Conflict	Scale	Input	10	Right	F10	F10
m2	15	<none>	Scale	Input	10	Right	F10	F10
m3	16	<none>	Scale	Input	10	Right	F10	F10
m4	17	<none>	Scale	Input	10	Right	F10	F10
m5	18	<none>	Scale	Input	10	Right	F10	F10
m6	19	<none>	Scale	Input	10	Right	F10	F10
m7	20	<none>	Scale	Input	10	Right	F10	F10
m8	21	<none>	Scale	Input	10	Right	F10	F10
m9	22	<none>	Scale	Input	10	Right	F10	F10
m10	23	<none>	Scale	Input	12	Right	F10	F10
m11	24	<none>	Scale	Input	12	Right	F10	F10
m12	25	<none>	Scale	Input	12	Right	F10	F10
m13	26	<none>	Scale	Input	12	Right	F10	F10
m14	27	<none>	Scale	Input	12	Right	F10	F10
m15	28	<none>	Scale	Input	12	Right	F10	F10
m16	29	<none>	Scale	Input	12	Right	F10	F10
m17	30	<none>	Scale	Input	12	Right	F10	F10
m18	31	<none>	Scale	Input	12	Right	F10	F10
m19	32	<none>	Scale	Input	12	Right	F10	F10

m20	33	<none>	Scale	Input	12	Right	F10	F10
m21	34	<none>	Scale	Input	12	Right	F10	F10
m22	35	<none>	Scale	Input	12	Right	F10	F10
m23	36	<none>	Scale	Input	12	Right	F10	F10
m24	37	<none>	Scale	Input	12	Right	F10	F10
m25	38	<none>	Scale	Input	12	Right	F10	F10
m26	39	<none>	Scale	Input	12	Right	F10	F10
m27	40	<none>	Scale	Input	12	Right	F10	F10
m28	41	<none>	Scale	Input	12	Right	F10	F10
m29	42	<none>	Scale	Input	12	Right	F10	F10
m30	43	<none>	Scale	Input	12	Right	F10	F10
m31	44	<none>	Scale	Input	12	Right	F10	F10
m32	45	<none>	Scale	Input	12	Right	F10	F10
m33	46	<none>	Scale	Input	12	Right	F10	F10
m34	47	<none>	Scale	Input	12	Right	F10	F10
m35	48	<none>	Scale	Input	12	Right	F10	F10
m36	49	<none>	Scale	Input	12	Right	F10	F10
m37	50	<none>	Scale	Input	12	Right	F10	F10
m38	51	<none>	Scale	Input	12	Right	F10	F10
m39	52	<none>	Scale	Input	12	Right	F10	F10
m40	53	<none>	Scale	Input	12	Right	F10	F10
m41	54	<none>	Scale	Input	12	Right	F10	F10
m42	55	<none>	Scale	Input	12	Right	F10	F10
n1	56	pregnancy concerns	Scale	Input	17	Right	F17	F17
n2	57	pregnancy concerns	Scale	Input	17	Right	F17	F17
n3	58	pregnancy concerns	Scale	Input	17	Right	F17	F17
n4	59	pregnancy concerns	Scale	Input	17	Right	F17	F17
n5	60	pregnancy concerns	Scale	Input	17	Right	F17	F17
n6	61	pregnancy concerns	Scale	Input	17	Right	F17	F17
n7	62	pregnancy concerns	Scale	Input	17	Right	F17	F17

n8	63	pregnancy concerns	Scale	Input	17	Right	F17	F17
n9	64	pregnancy concerns	Scale	Input	17	Right	F17	F17
n10	65	pregnancy concerns	Scale	Input	17	Right	F17	F17
n11	66	pregnancy concerns	Scale	Input	17	Right	F17	F17
n12	67	pregnancy concerns	Scale	Input	19	Right	F17	F17
s1	68	perceived stress	Scale	Input	23	Right	F23	F23
s2	69	perceived stress	Scale	Input	23	Right	F23	F23
s3	70	perceived stress	Scale	Input	23	Right	F23	F23
s4	71	perceived stress	Scale	Input	23	Right	F23	F23
s5	72	perceived stress	Scale	Input	23	Right	F23	F23
s6	73	perceived stress	Scale	Input	23	Right	F23	F23
s7	74	perceived stress	Scale	Input	23	Right	F23	F23
s8	75	perceived stress	Scale	Input	23	Right	F23	F23
s9	76	perceived stress	Scale	Input	23	Right	F23	F23
s10	77	perceived stress	Scale	Input	23	Right	F23	F23
s11	78	perceived stress	Scale	Input	23	Right	F23	F23
s12	79	perceived stress	Scale	Input	23	Right	F23	F23
type.of.delivery	80	<none>	Nominal	Input	12	Right	F12	F12
infantgender	81	<none>	Nominal	Input	8	Right	F8	F8
infantweight	82	<none>	Scale	Input	12	Right	F32.2	F32.2
breastfeeding	83	<none>	Nominal	Input	6	Right	F6	F6
milkpowder	84	<none>	Nominal	Input	6	Right	F6	F6
NICU	85	<none>	Nominal	Input	6	Right	F6	F6
e1	86	parenting competency	Scale	Input	12	Right	F28	F28
e2	87	<none>	Scale	Input	8	Right	F28	F28
e3	88	<none>	Scale	Input	8	Right	F28	F28
e4	89	<none>	Scale	Input	10	Right	F28	F28
e5	90	<none>	Scale	Input	8	Right	F28	F28
e6	91	<none>	Scale	Input	10	Right	F28	F28
e7	92	<none>	Scale	Input	9	Right	F28	F28
e8	93	<none>	Scale	Input	13	Right	F28	F28
e9	94	<none>	Scale	Input	10	Right	F28	F28

e10	95	<none>	Scale	Input	11	Right	F28	F28
e11	96	<none>	Scale	Input	10	Right	F28	F28
e12	97	<none>	Scale	Input	10	Right	F28	F28
e13	98	<none>	Scale	Input	11	Right	F28	F28
e14	99	<none>	Scale	Input	12	Right	F28	F28
e15	100	<none>	Scale	Input	11	Right	F28	F28
e16	101	<none>	Scale	Input	11	Right	F28	F28
e17	102	<none>	Scale	Input	14	Right	F28	F28

Variables in the working file

Variable Values

Value		Label
age	1	LOW20
	2	21-30
	3	31-40
	4	>40
job	1	Househod
	2	emploee
	3	student
	4	آself-employment
education	1	under diploma
	2	diploma
	3	bachelor
	4	Master and PhD
husband.age	1	<28
	2	29-33
	3	34-38
	4	>39
husband.education	1	LOWER 20 YEARS
	2	21-30
	3	31-40
	4	>40
husband.job	1	EMPLOEE
	2	MANUAL WORKER
	3	SELF EMPLOYMENT
	4	UNEMPLOYED
income	1	<4

	2	4-6
	3	6-8
	4	>8
marraige.lenght	1	<3
	2	3-6
	3	6-9
	4	>9
infertility	1	yes
	2	no
m1	1	always
	2	most of times
	3	some times
	4	rarely
	5	never
m2	1	always
	2	most of times
	3	some times
	4	rarely
	5	never
m3	1	always
	2	most of times
	3	some times
	4	rarely
	5	never
m4	1	always
	2	most of times
	3	some times
	4	rarely
	5	never
m5	1	always
	2	most of times
	3	some times
	4	rarely
	5	never
m6	1	always
	2	most of times
	3	some times
	4	rarely

	5	never
m7	1	always
	2	most of times
	3	some times
	4	rarely
	5	never
m8	1	always
	2	most of times
	3	some times
	4	rarely
	5	never
m9	1	always
	2	most of times
	3	some times
	4	rarely
	5	never
n1	0	not at all
	1	very littlee
	2	much
	3	very much
n2	0	not at all
	1	very littlee
	2	much
	3	very much
n3	0	not at all
	1	very littlee
	2	much
	3	very much
n4	0	not at all
	1	very littlee
	2	much
	3	very much
n5	0	not at all
	1	very littlee
	2	much
	3	very much
n6	0	not at all
	1	very littlee

	2	much
	3	very much
n7	0	not at all
	1	very littlee
	2	much
	3	very much
n8	0	not at all
	1	very littlee
	2	much
	3	very much
n9	0	not at all
	1	very littlee
	2	much
	3	very much
n10	0	not at all
	1	very littlee
	2	much
	3	very much
n11	0	not at all
	1	very littlee
	2	much
	3	very much
n12	0	not at all
	1	very littlee
	2	much
	3	very much
s1	1	strongly disagree
	2	disagree
	3	no idea
	4	agree
	5	strongly agree
s2	1	strongly disagree
	2	disagree
	3	no idea
	4	agree
	5	strongly agree
s3	1	strongly disagree
	2	disagree

	3	no idea
	4	agree
	5	strongly agree
s4	1	strongly disagree
	2	disagree
	3	no idea
	4	agree
	5	strongly agree
s5	1	strongly disagree
	2	disagree
	3	no idea
	4	agree
	5	strongly agree
s6	1	strongly disagree
	2	disagree
	3	no idea
	4	agree
	5	strongly agree
s7	1	strongly disagree
	2	disagree
	3	no idea
	4	agree
	5	strongly agree
s8	1	strongly disagree
	2	disagree
	3	no idea
	4	agree
	5	strongly agree
s9	1	strongly disagree
	2	disagree
	3	no idea
	4	agree
	5	strongly agree
s10	1	strongly disagree
	2	disagree
	3	no idea
	4	agree
	5	strongly agree

s11	1	strongly disagree
	2	disagree
	3	no idea
	4	agree
	5	strongly agree
s12	1	strongly disagree
	2	disagree
	3	no idea
	4	agree
	5	strongly agree
type.of.delivery	1	wd
	2	cs
infantgender	1	girl
	2	boy
breastfeeding	1	yes
	2	no
milkpowder	1	yes
	2	no
NICU	1	yes
	2	no
e1	1	strongly disagree
	2	somewhat disagree
	3	disagree
	4	some what agree
	5	agree
	6	strongly agree
e2	1	strongly disagree
	2	somewhat disagree
	3	disagree
	4	some what agree
	5	agree
	6	strongly agree
e3	1	strongly disagree
	2	somewhat disagree
	3	disagree
	4	some what agree
	5	agree
	6	strongly agree

e4	1	strongly disagree
	2	somewhat disagree
	3	disagree
	4	some what agree
	5	agree
	6	strongly agree
e5	1	strongly disagree
	2	somewhat disagree
	3	disagree
	4	some what agree
	5	agree
	6	strongly agree
e6	1	strongly disagree
	2	somewhat disagree
	3	disagree
	4	some what agree
	5	agree
	6	strongly agree
e7	1	strongly disagree
	2	somewhat disagree
	3	disagree
	4	some what agree
	5	agree
	6	strongly agree
e8	1	strongly disagree
	2	somewhat disagree
	3	disagree
	4	some what agree
	5	agree
	6	strongly agree
e9	1	strongly disagree
	2	somewhat disagree
	3	disagree
	4	some what agree
	5	agree
	6	strongly agree
e10	1	strongly disagree
	2	somewhat disagree

	3	disagree
	4	some what agree
	5	agree
	6	strongly agree
e11	1	strongly disagree
	2	somewhat disagree
	3	disagree
	4	some what agree
	5	agree
	6	strongly agree
e12	1	strongly disagree
	2	somewhat disagree
	3	disagree
	4	some what agree
	5	agree
	6	strongly agree
e13	1	strongly disagree
	2	somewhat disagree
	3	disagree
	4	some what agree
	5	agree
	6	strongly agree
e14	1	strongly disagree
	2	somewhat disagree
	3	disagree
	4	some what agree
	5	agree
	6	strongly agree
e15	1	strongly disagree
	2	somewhat disagree
	3	disagree
	4	some what agree
	5	agree
	6	strongly agree
e16	1	strongly disagree
	2	somewhat disagree
	3	disagree
	4	some what agree

	5	agree
	6	strongly agree
e17	1	strongly disagree
	2	somewhat disagree
	3	disagree
	4	some what agree
	5	agree
	6	strongly agree
